# Compound Matrix-Based Project Database (CMPD)

**DOI:** 10.1038/s41597-024-03154-x

**Published:** 2024-03-27

**Authors:** Zsolt T. Kosztyán, Gergely L. Novák

**Affiliations:** 1 Department of Quantitative Methods, University of Pannonia, Egyetem str. 10, Veszprém, H-8200 Hungary; 2 Continental Automotive Hungary Ltd., Házgyári str. 6-8., Veszprém, H-8200 Hungary

**Keywords:** Business, Technology

## Abstract

The impact of projects is vital, from business operations to research to the national economy. Therefore, management science and operation research have extensively studied project scheduling and resource allocation for over six decades. Project databases were proposed to test algorithms, including simulated or real, single or multiprojects, and single-mode or multi-mode projects. However, the dozens of project databases are extremely heterogeneous regarding the file structure and the features of the modeled projects. Furthermore, the efficiency and performance of project scheduling and resource allocation algorithms are susceptible to the characteristics of projects. Therefore, the proposed Compound Matrix-Based Project Database (CMPD) collects and consolidates the most frequently used project databases. The proposed Unified Matrix-Based Project-Planning Model (UMP) sparse matrix-based model enables the addition of new features to existing project structures, such as completion priorities, structural flexibility, and quality parameters, to broaden the scope of considered projects and to take account of flexible approaches, such as agile, extreme, and hybrid projects.

## Background & Summary

Overall, projects contribute almost 20% of a country’s GDP^1,2^. Therefore, for approximately six decades, management science and operations research has extensively studied project scheduling issues^3,4^. A novel project scheduling or resource allocation algorithm cannot be published until it is compared with other algorithms in existing project databases.

Most project databases are capable of storing (1) fixed^5,6^ project structures; (2) Different types of completion modes^7^, including (a) time demands and (b) resource demands; and (3) single^8^ and multiple^5^ project structures. In addition, several smaller project databases store data that can be assigned to resources rather than activities, for example, the use of skills^9^. However, these databases are not compatible with several others.

The main shortcomings of these databases are that (1) they are quite heterogeneous in terms of file structures and project characteristics^10^; (2) Important features, e.g., quality and priorities, are not included; (3) It is difficult to add data that cannot be directly linked to activities, e.g., skills, organizational hierarchies, responsibilities, etc.; (4) They completely neglect flexibility issues of the project, such as completion priorities and flexible dependencies.

To address this gap, we employed a recently published^10^ matrix-based UMP model that can store (1) single- and multimodal projects, (2) individual and multiprojects, and (3) fixed and flexible projects. In addition, features such as quality parameters, costs, and nonrenewable resources can be assigned to tasks as new domains (submatrices). This matrix approach allows further submatrices such as skills^11^ and maintainable system parameters^12^ to be specified. With the proposed parsers^13^, 12 existing, most frequently used project databases (including 23 datasets) are parsed into the proposed unified matrix-based project database, CMPD. The database includes not only single-mode but also multimode data, as well as single- and multiproject data. To validate the proposed CMPD, structural, time-related, and resource-related indicators are implemented^14^ to ensure adequate modeling of existing project structures in the proposed matrix-based database.

Project scheduling is an integral part of project management that involves the allocation of resources over time to perform a set of activities with dependencies. The classic resource-constrained project scheduling problem (RCPSP) and its extensions for multiple projects (RCMPSP) and multiple completion modes (MRCPSP) or both (MRCMPSP) are well known in the literature and are suitable for various practical scenarios. Recent extensions incorporating multiple skills^11^, flexible resource profiles^15^, task priorities, and flexible dependencies^10^ have gained significant attention. These advancements have highlighted the necessity of additional attributes in project scheduling models and the importance of model standardization^16^. Applications beyond projects and other industries could also benefit from the progression of new models^17,18^. For an overview of all problem variants and their characteristics, we refer to the survey of Hartmann and Briskorn^16^.

Project databases have long been studied in the project scheduling context, starting with the early Patterson^19^ set but constructed without well-defined problem parameters; subsequently, Boctor^20^ and other popular artificial databases, such as SMCP/SMFF^21^, PSPLIB^22^, RG^8,23^, and MMLIB^24^, play a significant role in benchmarking algorithms. A set of real-life project plans was also collected by Batselier *et al*.^6^. Databases containing multiple projects running in parallel were also established, including MPSPLIB^25^, BY^5^, RCMPSPLIB^26^, and MPLIB^7^. Some of the databases also support multiple completion modes (PSPLIB^22^, Boctor^20^, and MMLIB^24^). We refer to Table 1 for a list of the selected databases and their references, along with the number of existing and newly added instances.

**Table 1:** Summary of existing project databases, extended with flexible instances.

Projects	Databases	# Instances	# New instances
Single	Boctor^20^ https://www.om-db.wi.tum.de/psplib/dataob.html	240	12,480
	Kolisch^21^ https://github.com/novakge/project-parsers/tree/master/data	680	8,840
	MMLIB^24^ https://www.projectmanagement.ugent.be/sites/default/files/datasets/MMRCPSP/MMLIB.zip	4,320	294,840
	PAT^55^ https://www.om-db.wi.tum.de/psplib/dataob.html	110	1,430
	PSPLIB^22^ https://www.om-db.wi.tum.de/psplib/getdata_sm.html	13,222	461,448
	Real-life^6^ https://www.projectmanagement.ugent.be/sites/default/files/datasets/Empirical/DSLIB.zip	133	1,729
	RG^8^ https://www.projectmanagement.ugent.be/sites/default/files/files/datasets/AboutData.zip	2,280	29,640
Multiple	BY^5^ https://www.projectmanagement.ugent.be/sites/default/files/datasets/RCMPSP/BY.zip	12,320	160,160
	MPLIB1^7^ https://www.projectmanagement.ugent.be/sites/default/files/datasets/RCMPSP/MPLIB.zip	4,550	59,150
	MPLIB2^56^ https://www.projectmanagement.ugent.be/sites/default/files/datasets/RCMPSP/MPLIB.zip	35,085	456,105
	MPSPLIB^25^ http://www.mpsplib.com/data/mp_all.zip	140	1,820
	RCMPSPLIB^26^ https://www.projectmanagement.ugent.be/sites/default/files/datasets/RCMPSP/RCMPSPLIB.zip	26	338

The PSPLIB dataset is still considered the most popular dataset in recent RCPSP literature^27^. A survey^28^ considering the RCMPSP variant highlighted the MPSPLIB dataset as the most commonly used benchmark set.

There are other databases that mostly target different RCPSP variants or candidates for later release. We reviewed only the most important studies without a complete list, which is outside the scope of this paper. The MT dataset^29^ is mainly used for schedule risk analysis and earned value management and contains project structures that can be combined with additional resource data; this dataset is called ResSet, which results in the NetRes dataset^30^. DC1^31^ and DC2^32^ are studied within the context of the RCPSP with discounted cash flows. The CV set^33^ and the sD set^27^ contain RCPSP instances that are difficult to solve. MISTA2013^34^ is a dataset and generator for the multimode resource-constrained multiple project scheduling problem (MRCMPSP) and combines instances from the PSPLIB. The BL^35^ and PACK^36^ datasets are also modifications of the PSPLIB and were designed for the context of highly disjunctive and cumulative scheduling of RCPSP, respectively. The AT dataset^37^ was one of the early sets generated with well-defined problem parameters. The ASLIB^38^ dataset contains instances for the resource-constrained project scheduling problem with alternative subgraphs (RCPSP-AS). The MSLIB and SSLIB^39^ databases were proposed for the multiskilled resource-constrained project scheduling problem (MSRCPSP). The RACP30^40^ dataset was proposed in the context of the resource availability cost problem (RACP).

Most of the existing databases and available methods support only a fixed logic plan or consider a limited number of scheduling alternatives^4,17,41–45^. This approach is intuitive for traditional project management methods, which aim to minimize changes and follow rigid project plans^46,47^. However, agile, hybrid, and extreme project management methods address uncertainty by frequently adapting task priorities and dependencies^48,49^. To overcome the limitations of fixed project plans and to support the features of emerging project management approaches, the Flexible Structures Generator (FSG) enables the respecification of task priorities and dependencies, allowing existing project structures to be flexible. As a result, existing project databases can be extended with both traditional and flexible project structures for further research.

## Methods

The database comprises 12 libraries, 23 datasets, and 73,106 instances. An additional 1,561,086 flexible instances were generated using the FSG method. The original databases were collected via a thorough literature review process conducted by the authors, targeting databases of the popular (multimode) resource-constrained (multi)project scheduling problem types, (M)RC(M)PSP. As a result, additional data sources were identified and collected, broadening the list mentioned in existing surveys^28,50^. To maintain data quality, relevant academic papers in project management and scheduling were selected to support the database’s integrity and reliability. Some less popular datasets have already been collected and are under preparation for intended future releases.

### The unified model for storing project data instances

**Fig. 1:**
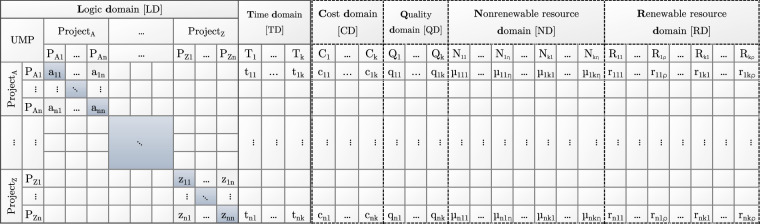
Structure of the unified matrix-based project planning model (UMP).

The proposed *unified matrix-based project planning model* (UMP) can represent all features of widely accepted databases, i.e., individual and multiple projects, single and multimodal completions, and renewable and nonrenewable resources. It contains two mandatory and four supplementary domains (marked with dashed lines), as shown in Fig. 1.

**LD** The logic domain is an *n* by *n* matrix, where *n* is the number of tasks. Each cell contains a value in the interval [0,1].

**TD** The time domain is an *n* by *k* matrix with positive real values, where *k* is the number of completion modes.

The first mandatory domain is the logic domain, ${\bf{LD}}\in {[0,1]}^{n\times n}$. The diagonal values in **LD** represent the task priority values. If the diagonal value is 0, the task will not be completed; if the diagonal value is 1, the task is mandatory. If the diagonal value is between 0 and 1, the task is supplementary, indicating that, depending on the decision, it will be either completed or omitted/postponed. The out-diagonal values represent the dependencies between tasks or projects (programs).

The additional supplementary domains are as follows:

**CD** The cost domain is an *n* by *k* nonnegative matrix of the task costs

**QD** The quality domain is an *n* by *k*, nonnegative matrix of the task quality parameters, where the quality parameters are in [0,1]

**ND** The nonrenewable resource domain is an *n* by *k η* nonnegative matrix of nonrenewable resource demands, where *η* is the number of types of nonrenewable resources

**RD **The renewable resource domain is an *n* by *k ρ* nonnegative matrix of renewable resource demands, where *ρ* is the number of types of renewable resources

The proposed model thus enables the representation of various projects and features, including flexibility.

### Generating flexible structures

Four types of structures are generated for each flexibility level. *The maximal* structures are the equivalents of the original instances. In the case of *minimal* structures, all flexible dependencies and supplementary tasks are excluded; for *minimax*, all supplementary tasks with flexible dependencies are removed; and for *maximin* structures, only their flexible dependencies are removed.

**Fig. 2:**
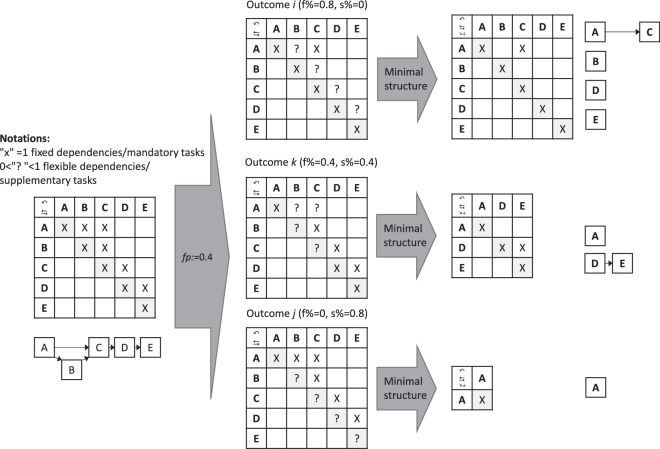
Example of generating flexible and minimal structures.

An example of the construction process of flexible structures from existing instances is shown in Fig. 2 for *minimal* structures.

The left side of Fig. 2 shows the original logic domain: the flexibility parameter (*fp*) is set to 0.4 in this case. In the first step, fixed dependencies/mandatory tasks (denoted by the “X” symbol) become flexible (denoted by “? ”, where “?” indicates a value between 0 and 1). The right side of Fig. 2 shows the minimal structure of the project. The center of Fig. 2 shows three possible outcomes from $$\left(\begin{array}{c}10\\ 4\end{array}\right)$$. Because the number of “X” symbols is 10, we have *fp* = 0.4. Outcome *i* retains all tasks but cuts almost all dependencies, while outcome *j* retains only one task from the original project. In the general case, several dependencies are cut, and several tasks are omitted, e.g., in outcome *k*. The FSG algorithm has several steps. It processes project instances by iterating through all directories and loading the necessary input variables. For each fixed task *l*_*ii*_ = 1 and all fixed dependencies *l*_*ij*_ = 1, ($i\ne j$) in the logic domain (LD), a matrix with uniform random values *rv*_*ij*_ from the range of [0,1] is generated. In the next step, these values are evaluated depending on the type of structure for the given flexibility parameter (*fp*):

**maximal (original)**: All tasks and dependencies are retained, and *fp* is set to 0:

1 $${l}_{ij}^{\max }=1$$

**maximin**: tasks are retained, and dependencies are updated:

2 $${l}_{ij}^{{\rm{maximin}}}=\left(\begin{array}{l}\lceil r{v}_{ij}\rceil {\rm{if}}\,i=j\,{\rm{and}}\,r{v}_{ij}\le fp,\\ \lfloor r{v}_{ij}\rfloor {\rm{if}}\,i\ne j\,{\rm{and}}\,r{v}_{ij}\le fp,\\ 0\,{\rm{otherwise}}\end{array}\right.$$

**Minimax**: dependencies are kept, and tasks are updated:

3 $${l}_{ij}^{{\rm{minimax}}}=\left(\begin{array}{l}\lfloor r{v}_{ij}\rfloor {\rm{if}}\,i=j\,{\rm{and}}\,r{v}_{ij}\ge fp,\\ \lceil r{v}_{ij}\rceil {\rm{if}}\,i\ne j{\rm{,}}\lfloor r{v}_{ii}\rfloor =\lfloor r{v}_{jj}\rfloor =1\,{\rm{and}}\,r{v}_{ij}\le fp,\\ 0\,{\rm{otherwise}}\end{array}\right.$$

**minimal**: tasks and dependencies are replaced

4 $${l}_{ij}^{\min }=\lfloor r{v}_{ij}\rfloor ,{\rm{if}}\;r{v}_{ij}\le fp,$$

where ${l}_{ij}^{\max },{l}_{ij}^{\mathrm{maximin}},{l}_{ij}^{\mathrm{minimax}},{l}_{ij}^{\min },$ are the (*i, j*) cells of the logic domains of the maximal (original), maximin, minimal, and minimax structures, respectively, with $i,j=1,2,..,n$. The $\lceil \cdot \rceil$ ($\lfloor \cdot \rfloor$) operators denote the rounding up (rounding down) of real numbers to the closest integer. The resulting flexible structures are saved in a designated directory. The random seed of the pseudorandom number generator was fixed for reproducibility. The various structure types add backward compatibility and provide a connection between traditional and flexible project plans and approaches.

## Data Records

Since the data originate from the reviewed academic literature, redundancy and quality concerns are mitigated. The database incorporates data from various sources and formats by employing the described unified model. Table 2 lists the main characteristics of the selected databases.

**Table 2:** Summary of all supported databases and their main attributes.

Dataset	Set(s)	Instances	# Tasks	# Projects	Type	Method	Mode(s)	Attributes	File extension(s)	Generator
Boctor^20^	Boctor50	120	50	Single	Artificial	Generated	Multi (4)	Time, renewable resources	*.prb	Unpublished
	Boctor100	120	100							
Kolisch^21^	SMCP	200	10, 20, 30, 40	Single	Artificial	Generated	Single	Time, renewable resources	*.rcp	ProGen^21^
	SMFF	480	30							
MMLIB^24^	MMLIB50	540	50	Single	Artificial	Generated	Multi (3)	Time, re/non-renewable resources	*.mm	RanGen1^52^
	MMLIB100	540	100							
	MMLIB+	3,240	50, 100				1*Multi (9)			
PAT^19^	Patterson	110	5–49	Single	Artificial	Generated	Single	Time, renewable resources	*.prb	Unpublished
PSPLIB^22^	SM [j]	2,040	30, 60, 90, 120	Single	Artificial	Generated	Single	Time, re/non-renewable resources	*.sm	ProGen
	MM [c,j,m,n,r]	11,182	10, 12, 16, 20, 30	Single	Artificial	Generated	Multi (1–5)	Time, re/non-renewable resources	*.mm	ProGen
Real-life^6^	—	133	26–151	Single	Empirical	Collected	Single	Time, cost, re-, non-renewable resources	*.p2x	—
RG30^8^	Set1	900	30	Single	Artificial	Generated	Single	Time, renewable resources	*.rcp	RanGen2^8^
	Set2	180	30							
	Set3	240	30							
	Set4	240	30							
	Set5	240	30							
RG300^8^	—	480	300	Single	Artificial	Generated	Single	Time, renewable resources	*.rcp	RanGen1
BY^5^	Rep1-Rep20	12,320	20	Multi (3)	Artificial	Generated	Single	Time, cost, renewable resources	*.xlsm (*.rcmp)	MNPGbrowning2010random
MPLIB1^7^	Set1	833	360	Multi (6)	Artificial	Generated	Single	Time, renewable resources	*.rcmp	Modified MNPG
	Set2	1,463	720	Multi (12)						
	Set3	2,254	1,440	Multi (24)						
MPLIB2^56^	Set1	10,125	500, 1,000	Multi (10, 20, 30)	Artificial	Generated	Single	Time, renewable resources	*.rcmp	Modified MNPG
	Set2	8,640	500, 1,000, 1,500	Multi (10, 20, 30)						
	Set3	8,640	1,000	Multi (20)						
	Set4	7,680	1,000	Multi (20)						
MPSPLIB^25^	—	140	60–2,400	Multi (2, 5, 10, 20)	Artificial	Generated, combined	Single	Time, renewable resources	*.sm, *.xml (*.rcmp)	Various (ProGen / unpublished)
RCMPSPLIB^26^	—	26	60–450	Multi (2–10)	Artificial	Generated, combined	Single	Time, renewable resources	*.txt (*.rcmp)	Various (ProGen, MNPG, unpublished)

Data profiling was conducted for each database format through examination. None of the databases showed interpretation issues or a lack of extractable data. The methodologies employed by the original authors in generating or collecting the databases were studied in advance to understand the characteristics, methodology, and assumptions of their data. The original data were assessed for important quality characteristics, such as accuracy, consistency, completeness, and currency^51^. Additional consistency checks were executed in the preprocessing phase, ensuring that no contradictory conclusions could be drawn from the original data. Each instance contains descriptive information that can be recalculated from the data itself. These variables are the number of activities and the number of (non)renewable resources. In addition, logical rules can be directly applied for verification and to identify possible conflicts within the data. The number of (non)renewable resources is directly related to the dimension of the constraint vector, while the number of columns in the resource and cost vectors increases proportionally with the number of available modes. Some instances contain the number of precedences or the critical path length, which can be calculated from task precedences and durations. The topological ordering of the logic network, including testing for a lack of cycles in the graphs, was also verified during the process. In the case of generated data, the designed parameter ranges described in the original papers were cross-checked with the help of indicators. Outliers were assessed as individual cases through a detailed examination of the localized data. No missing entries or other anomalies were identified in any of the instances.

To seamlessly integrate diverse data into our model, automated scripts are employed. The necessary conversions or transformations are automatically performed by the developed toolset, which is provided as part of the repository. The provided scripts are designed to interpret and extract all possible attributes and information from each original dataset, ensuring reliable and reproducible data transformation. Format descriptors are collected at the code repository under the ‘docs’ folder. Instances generated by standard project generators, such as ProGen^21^ and RanGen 1^52^ and 2^8^, of the collected datasets are also supported by the parser. For convenient access to the released version of the CMPD, including flexible instances, please refer to the deposit at Figshare^53^. For databases containing a significant number of files or larger datasets, users can generate instances on their local computers, provided they meet the required hardware and software prerequisites.

**Fig. 3:**
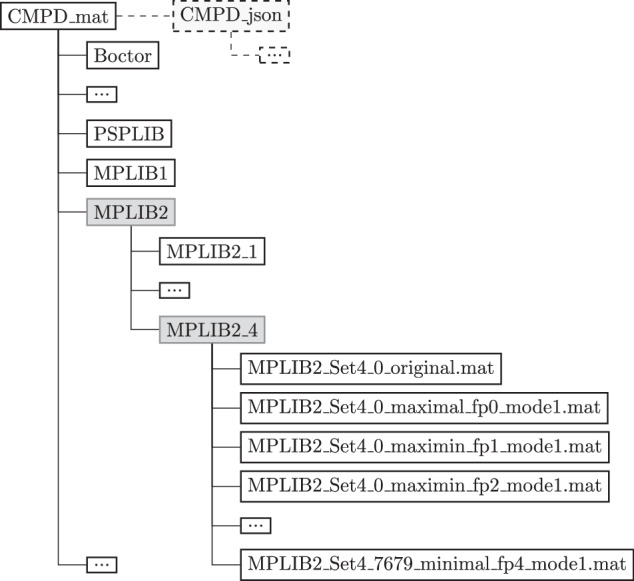
Database directory structure and filenames.

The CMPD reflects library and dataset folder names similar to those in the literature within its folder structure. To distinguish the new output format, instances are converted and saved using a predefined naming convention. Each folder contains the standardized output format of the original and flexible instances as MAT files, ensuring consistency. The example folder structure and filenames are shown in Fig. 3.

The libraries are stored in the *CMPD_mat* folder, and *CMPD_json* mirrors it in the widely adopted JSON format. Data libraries can have multiple datasets as subfolders, containing instances as separate files. The naming convention for flexible instances follows the pattern: CMPD_<format>\<library>\<dataset>\<instance#>_<structure_type>_fp<#>_mode<#>.<extension>, where the type of structure can be one of {maximal,maximin,minimax,minimal}; the ‘mode’ specifies the execution mode of a particular instance; and ‘fp’ is the flexibility parameter in the range of {0,1,2,3,4}, used to generate the instance, and the extension is either “.mat” or “.json”. For the sake of completeness, the original instances are also saved without the ‘fp’ and ‘mode’ suffices.

## Technical Validation

To ensure the accuracy, reliability, and consistency of the data, several actions were taken. Unit tests were created during the development and verification process to verify the functionality of the data conversion and generation. The data consistency was checked with an automated test suite ensuring that all the instances conformed to the defined data dictionary provided in Table 3.

**Table 3:** Data dictionary for all CMPD instances.

Name	Type	Min. size [row,col.]	Max size [row,col.]	Value range
constr	double	[1, 3]	[1, Inf]	[−1, Inf]
sim_type	double	[1, 1]	[1, 1]	[1, 3]
struct_type	string	[1, 1]	[1, 1]	[0, 8]
release_dates	double	[1, 1]	[1, Inf]	[0, Inf]
num_projects	double	[1, 1]	[1, 1]	[1, Inf]
num_activities	double	[1, 1]	[1, Inf]	[1, Inf]
num_r_resources	double	[1, 1]	[1, Inf]	[0, Inf]
num_modes	double	[1, 1]	[1, 1]	[1, Inf]
PDM	double	[1, 1]	[Inf, Inf]	[0, Inf]
mode	double	[1, 1]	[1, 1]	[0, Inf]
fr	double	[1, 1]	[1, 1]	[−1, 1]
sr	double	[1, 1]	[1, 1]	[−1, 1]
fp	double	[1, 1]	[1, 1]	[0.0, 0.4]

The test cases are designed to follow an incremental approach, starting with generic tests, such as checking the folder structure, size and number of files, and adherence to naming conventions. Equivalent tests are further executed on the level of variables, extended with specific cases for variable type, size, invalid or missing entries, and value ranges, according to the provided metadata. The logical relationships between variables are also tested. The matrices and submatrices were verified for size definitions given by the UMP. Possible errors, including exceptions, were handled by either the built-in software libraries or additionally implemented by design. Interactive debugging sessions and fault injection techniques were used to identify any potential exceptions in the parsing process for the different formats.

Reviews were also conducted to check the quality and integrity of the data. Project-related indicators were also used to assess the equivalence of the original and converted data and to compare them with the results from the literature. Subsequent generations of the database were compared to ensure reproducibility on both the Unix and Windows platforms. In addition, joint reviews by experts and paired programming were applied during the development process.

**Fig. 4:**
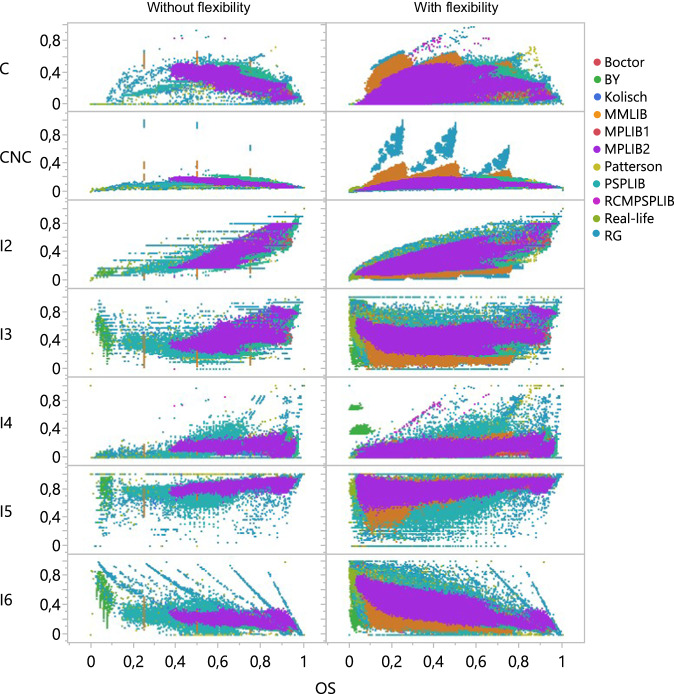
Topological feature space of all databases concerning flexibility.

Extensive statistical analyses and comparisons between the datasets were performed to validate the data. These analyses provided an understanding of each dataset’s common and unique characteristics. All the databases were checked for the coverage of numerous indicators using scatterplots. Figure 4 shows an example of the comparison between different network-related indicator values for the original and flexible structures. We refer to Kosztyán *et al*.^10^ for a detailed description of the applied indicators. The order strength (OS) indicator provided the most uniform coverage of values and was therefore selected for the horizontal axis, while the complexity of network coefficient (CNC) indicator was normalized to the [0,1] range for comparison. Databases such as MPLIB, MMLIB, and RG dominate all feature spaces, while BY covers a smaller but unique area. PSPLIB shows relatively good coverage even without introducing flexibility. Complexity decreased with flexibility, as indicated by C and CNC, bringing value to lower regions, and the seriality of task execution (I2) decreased. In general, the new flexible structures widened the indicator ranges and provided a more diverse set of values that have never been tested by project scheduling and resource allocation algorithms before.

The article^10^ associated with the dataset discusses the main results and findings of further evaluations. During the validation process, potential sources of errors, such as formatting differences or missing data entries, were considered and addressed to ensure the validity and reliability of the dataset.

## Usage Notes

By loading the database in MATLAB or an open-source alternative, the GNU Octave^54^ environment is straightforward, as determined by using either the drag&drop functionality or the built-in ‘load’ function. The data instances are stored as “.MAT” container or “.JSON” formatted files, each containing the following minimum set of standardized variables:

- *PDM*: This variable contains a matrix with specific domains available for the instance.
- *num_activities*: This variable represents the number of activities in a project. A multiproject is a vector of activity numbers for each project.
- *num_r_resources*: This variable represents the number of renewable resource types.
- *constr*: This variable stores the constraints set for the particular instance.

The instances might contain other optional variables depending on the applicability and actual content. For example, ‘*fp*’ stores the flexibility parameter used by FSG, while ‘*num_modes*’ indicates the number of execution modes available for the original instance. A detailed view of all the variables and their attributes that are stored in the instances is given in Table 4.

**Table 4:** Variables and their attributes within an instance.

Example: “CMPD_mat\PSPLIB\j30sm\j301_1_NTP_maximal_fp0_mode1.mat”				
Variable name	Class	Size	Bytes	Description
PDM	double	30 × 36	8640	Project Domain Matrix [LD,TD,{CD,QD,ND,RD}]
num_activities	double	1 × 1	8	Number of tasks
num_projects	double	1 × 1	8	Number of projects
num_r_resources	double	1 × 1	8	Number of (renewable) resources
num_modes	double	1 × 1	8	Number of completion modes (1,…,w)
fp	double	1 × 1	8	Flexibility parameter {0,0.1,0.2,0.3,0.4}
fr	double	1 × 1	8	Rate of flexible dependencies [−1, 1]
sr	double	1 × 1	8	Rate of supplementary tasks [−1, 1]
mode	double	1 × 1	8	Selected completion mode (0,…,w)
constr	double	1 × 5	40	Constraints vector [*C*_*time*_, *C*_*cost*_,{*C*_*quality*_}, {*C*_*resource*_}, *C*score]
release_dates	double	1 × 1	8	Earliest allowed start time of projects
struct_type	string	1 × 1	150	Type of structure {original,maximal,maximin,minimax,minimal}

Once the instances are loaded in the workspace, variables can be accessed using their respective names, or it is also possible to access and change variables in the MAT files without loading them into memory.

If necessary, the MAT files can be manipulated and saved during the research process. Additionally, it is possible to extend the database with calculated indicator values, providing additional data to work with. The database itself is designed to ease future expansions, enabling the inclusion of new libraries, datasets, and instances. The structured nature of the database enables easy versioning, which can be managed through the popular GitHub platform and MathWorks site. To ensure the integrity of future updates and prevent any negative impacts or regressions, automated unit tests and use cases are implemented as part of the maintenance process. Users can run all available tests using the *‘runtests’* command executed in the source code folder. The source files and original databases are securely stored and made accessible through a public GitHub repository. Any academic or professional contributions to the repository and database management are handled within the GitHub platform, which facilitates discussion, issue reporting, and pull request processes and is maintained by key users.

## Data Availability

The source code is tracked in the Git versioning system and can be publicly accessed from the repository at https://github.com/novakge/project-parsers and https://github.com/novakge/project-indicators without registration. It is licensed under the terms GNU General Public License v3.0. A runnable (reproducible) code capsule can be found at Code Ocean. The code is tested against MATLAB R2020a or later releases with the Global Optimization Toolbox. A developer manual, including examples, is located in the repository’s Readme file.
